# The Integrative Analysis of Competitive Endogenous RNA Regulatory Networks in Coronary Artery Disease

**DOI:** 10.3389/fcvm.2021.647953

**Published:** 2021-09-22

**Authors:** Yuyao Ji, Tao Yan, Shijie Zhu, Runda Wu, Miao Zhu, Yangyang Zhang, Changfa Guo, Kang Yao

**Affiliations:** ^1^Department of Cardiology, Zhongshan Hospital, Shanghai Institute of Cardiovascular Diseases, Fudan University, Shanghai, China; ^2^Department of Cardiovascular Surgery, Zhongshan Hospital, Fudan University, Shanghai, China; ^3^Department of Cardiovascular Surgery, Shanghai East Hospital, Tongji University School of Medicine, Shanghai, China

**Keywords:** coronary artery disease, non-coding RNA, ceRNA, bioinformatics, WGCNA

## Abstract

**Background:** Coronary artery disease (CAD) is the leading cause of cardiovascular death. The competitive endogenous RNAs (ceRNAs) hypothesis is a new theory that explains the relationship between lncRNAs and miRNAs. The mechanism of ceRNAs in the pathological process of CAD has not been fully elucidated. The objective of this study was to explore the ceRNA mechanism in CAD using the integrative bioinformatics analysis and provide new research ideas for the occurrence and development of CAD.

**Methods:** The GSE113079 dataset was downloaded, and differentially expressed lncRNAs (DElncRNAs) and genes (DEGs) were identified using the limma package in the R language. Weighted gene correlation network analysis (WGCNA) was performed on DElncRNAs and DEGs to explore lncRNAs and genes associated with CAD. Functional enrichment analysis was performed on hub genes in the significant module identified *via* WGCNA. Four online databases, including TargetScan, miRDB, miRTarBase, and Starbase, combined with an online tool, miRWalk, were used to construct ceRNA regulatory networks.

**Results:** DEGs were clustered into ten co-expression modules with different colors using WGCNA. The brown module was identified as the key module with the highest correlation coefficient. 188 hub genes were identified in the brown module for functional enrichment analysis. DElncRNAs were clustered into sixteen modules, including seven modules related to CAD with the correlation coefficient more than 0.5. Three ceRNA networks were identified, including OIP5-AS1-miR-204-5p/miR-211-5p-SMOC1, OIP5-AS1-miR-92b-3p-DKK3, and OIP5-AS1-miR-25-3p-TMEM184B.

**Conclusion:** Three ceRNA regulatory networks identified in this study may play crucial roles in the occurrence and development of CAD, which provide novel insights into the ceRNA mechanism in CAD.

## Introduction

Cardiovascular disease (CVD) is one of the leading causes of death in the world. Coronary artery disease (CAD) is the main cause of cardiovascular death (1), which increases the morbidity, mortality, and economic burden on societies worldwide (2). The occurrence of CAD is associated with the interplay of genetic and environmental factors (3). Diabetes, hypertension, obesity, and smoking are significant risk factors for CAD (4, 5). Vascular stenosis caused by CAD is the main cause of coronary atherosclerosis and ischemia. The sclerotic plaques are at risk of rupture, leading to myocardial infarction and eventually death (4, 5). Although considerable efforts have been made, it remains a daunting task to prevent and cure CAD. Therefore, further research is urgently needed to understand its pathophysiological process.

Non-coding RNA (ncRNA), including lncRNA, circRNA, and miRNA, is a kind of RNA that does not encode proteins, which plays an essential role in the occurrence and development of CAD. LncRNAs are transcripts with a length of >200 nucleotides participating in a variety of critical biological processes (6). It was reported that lncRNAs play a significant role in the core stages of CAD, including lipid metabolism, inflammation, vascular cell proliferation, apoptosis, adhesion and migration, and angiogenesis (7). The competitive endogenous RNAs (ceRNAs) hypothesis is a theory that explains the relationship between lncRNAs and miRNAs. In this hypothesis, lncRNAs rich in miRNA binding sites can bind miRNAs and act as a miRNA sponge, leading to changes in expression levels of miRNA-target genes (8, 9). Although a growing body of evidence demonstrated that ncRNAs were associated with the development of CAD, research on the role of ceRNAs in the pathological process of CAD is still insufficient. Therefore, further efforts are warranted to elucidate the ceRNA mechanism in CAD.

In this study, we aimed to explore the complex interaction between lncRNAs, miRNAs, and mRNAs to investigate the potential mechanism of ceRNAs in CAD. We performed weighted gene correlation network analysis (WGCNA) on the GSE113079 to screen out lncRNAs and mRNAs associated with CAD. Then several online databases and tools were used to construct the ceRNA regulatory networks. We hope this study can provide potential targets and new research ideas for understanding the ceRNA mechanism in CAD.

## Methods and Materials

### Data Processing

The GSE113079 dataset was downloaded from the Gene Expression Omnibus (GEO) database. The dataset was based on the GPL20115 platform (Agilent-067406 Human CBC lncRNA + mRNA microarray V4.0), containing 141 samples of peripheral blood mononuclear cells (PBMCs) in 93 patients with CAD and 48 healthy controls. The R language was applied to process the dataset. The Linear Models for Microarray data (limma), a package in the R language, was used to identify differentially expressed genes (DEGs) and differentially expressed lncRNAs (DElncRNAs) with the cut-off point of adj. *p* < 0.05.

### WGCNA

DEGs identified by the limma package were imported for WGCNA to construct the gene co-expression network. First, the correlation network was constructed with an appropriate soft-thresholding power β realizing the scale-free topology criterion of *R*^2^ > 0.85. Second, the average linkage hierarchical clustering method was applied to cluster DEGs into different modules with different colors. The threshold for module merging was set as 0.25, and the minimum number of genes in each module was thirty. Hub genes correlation threshold was 0.9. Third, the correlation between each module and CAD was calculated using Pearson's correlation method. The module with a *p* < 0.05 and the highest correlation coefficient was screened out for further analysis. Similarly, DElncRNAs were also imported for WGCNA using the same parameters and procedures.

### Functional Enrichment

The Database for Annotation, Visualization and Integrated Discovery (DAVID, v6.8) was used to perform the Gene Ontology (GO) enrichment analysis, which revealed the biological processes (BPs), cellular components (CCs), and molecular functions (MFs) related to hub genes in the module identified above. GO terms with a *p* < 0.05 were considered significant enrichment. Metascape, a powerful online tool for gene function annotation, was also applied for functional enrichment.

### Construction of the ceRNA Network

The online tool miRWalk applies a machine-learning algorithm to predict miRNA-target interactions, including those that have been validated experimentally. Three online databases, including TargetScan, miRDB, and miRTarBase, combined with miRWalk, were used for the prediction of target miRNAs to ensure the robustness of the interactions between miRNAs and hub genes identified above. The Starbase is a public database that can search for potential miRNA-lncRNA interactions through high-throughput data. It was used to predict the relationships between target miRNAs and DElncRNAs to identify potential interactions. LncRNA-miRNA-mRNA regulatory network was visualized utilizing the Cytoscape software (v3.8.1).

### Quantitative Real-Time PCR (qRT-PCR)

Ten patients with CAD and ten without CAD confirmed by coronary angiography were included in this study. This study was in full compliance with the Declaration of Helsinki and approved by the Medical Ethics Committee of Shanghai Tenth People's Hospital, Tongji University. Written informed consent was obtained from all subjects participating in this study. Blood samples were collected and total RNA was extracted following the manufacturer's instruction (QIAGEN, Frankfurt, Germany). Briefly, mix one volume of whole blood with five volumes of buffer in an eppendorf tube. After incubating for 15 min on ice, centrifuge at 3,000 rpm for 10 min at 4°C and discard supernatant. Transfer lysate to spin column to centrifuge at 14,000 rpm and pipet 50 μl of RNase-free water. The Complementary DNA (cDNA) was synthesized by reverse transcription at 42°C for 60 min and then at 95°C for 5 min with the PrimeScript^TM^ RT reagent Kit (Takara, Otsu, Japan). TB Green® Premix Ex Taq^TM^ II (Takara, Otsu, Japan) was applied to perform qRT-PCR at the temperature of 95°C for 30 s, followed by 40 cycles with the temperature of 95°C for 5 s and 60°C for 34 s on QuantStudio^TM^ 5 System (Thermo Fisher Scientific, Waltham, MA, USA). The expression of RNA levels was normalized by GAPDH and U6, and the 2^−ΔΔCT^ method was applied to calculate the relative expression levels. All sequences for RNA primers (Sangon Biotech, Shanghai, China) are shown in Table 1.

**Table 1:** RNA primer sequences for quantitative real-time PCR.

**RNA**	**Sequences**
OIP5-AS1	Forward: CCACCACGCTCAGCCTGATTTC
	Reverse: TTTCCACGATGACCCAACCACAAG
DKK3	Forward: ACGAGTGCATCATCGACGAG
	Reverse: GCAGTCCCTCTGGTTGTCAC
SMOC1	Forward: TCAGGTTCAGTCACCGACAAG
	Reverse: TCCTGGTCACACGAATAGACTT
TMEM184B	Forward: ACTACGTGTACTTCGGCACC
	Reverse: CTGGACTCAATGGGTTTTCCTC
GAPDH	Forward: GGAGCGAGATCCCTCCAAAAT
	Reverse: GGCTGTTGTCATACTTCTCATGG
miR-204-5p	Forward: CGCGTTCCCTTTGTCATCCT
	Reverse: AGTGCAGGGTCCGAGGTATT
	RT: GTCGTATCCAGTGCAGGGTCCGAGGTATTCGC
	ACTGGATACGACAGGCAT
miR-211-5p	Forward: CGCGTTCCCTTTGTCATCCT
	Reverse: AGTGCAGGGTCCGAGGTATT
	RT: GTCGTATCCAGTGCAGGGTCCGAGGTATTCGC
	ACTGGATACGACAGGCGA
miR-92b-3p	Forward: GCGTATTGCACTCGTCCCG
	Reverse: AGTGCAGGGTCCGAGGTATT
	RT: GTCGTATCCAGTGCAGGGTCCGAGGTATTCGCAC
	TGGATACGACGGAGGC
miR-25-3p	Forward: GCGCATTGCACTTGTCTCG
	Reverse: AGTGCAGGGTCCGAGGTATT
	RT: GTCGTATCCAGTGCAGGGTCCGAGGTATTCGCACTGGA
	TACGACTCAGAC
U6	Forward: AGAGAAGATTAGCATGGCCCCTG
	Reverse: ATCCAGTGCAGGGTCCGAGG
	RT: GTCGTATCCAGTGCAGGGTCCGAGGTATTCGCA
	CTGGATACGACAAAATA

## Results

### Identification of DEGs and DElncRNAs

A total of 20,128 DElncRNAs, including 6,103 upregulated and 14,025 downregulated, which were differentially expressed between CAD samples and healthy controls, were identified in the GSE113079 dataset with the limma package. And 11,487 DEGs were identified, including 5,993 upregulated and 5,494 downregulated.

### WGCNA

**Figure 1:**
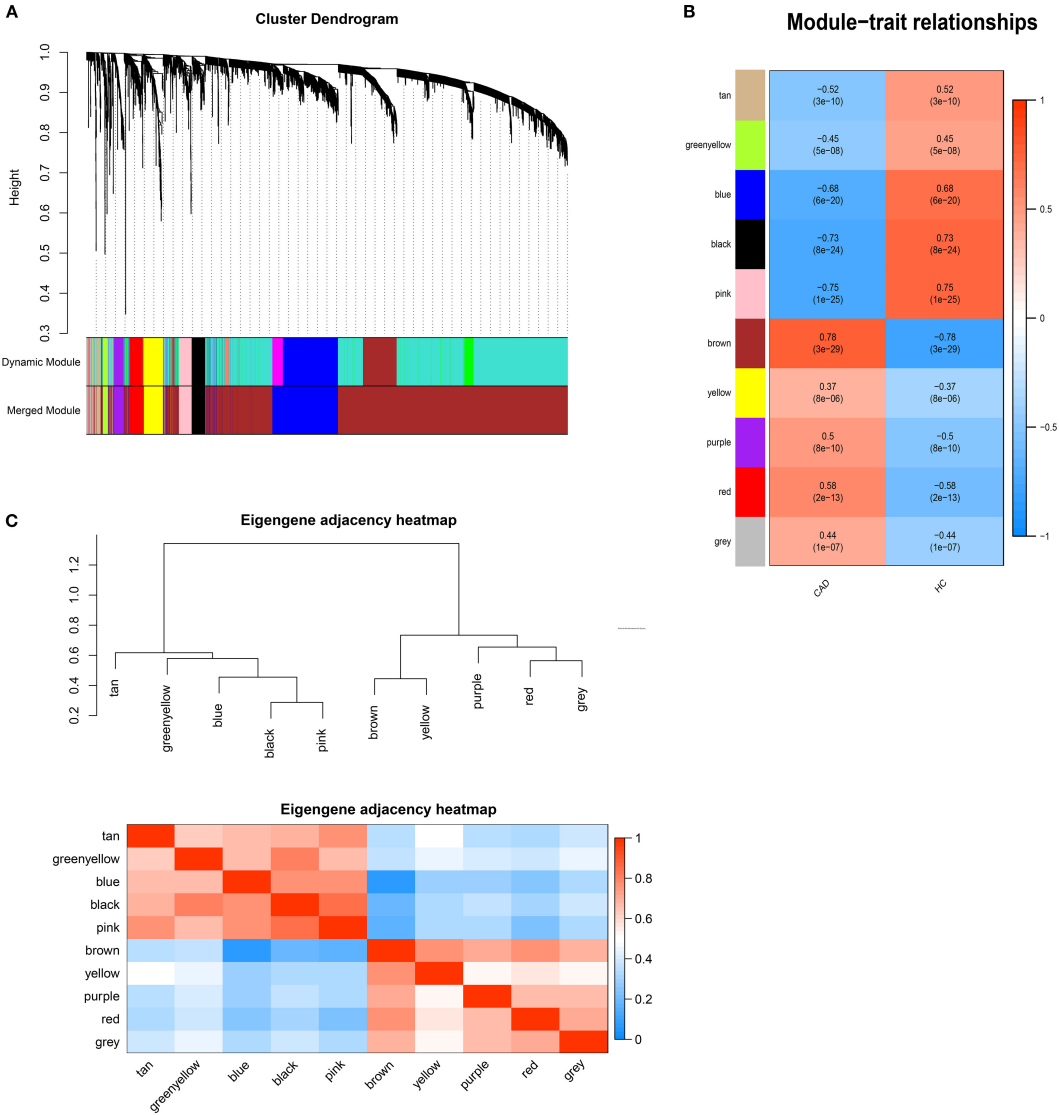
Weighted genes correlation network analysis. **(A)** The dendrogram of differentially expressed genes. **(B)** The heatmap of module-trait correlations. Blue represents negative correlation, and red represents positive correlation. **(C)** The clustering heatmap between modules. Red means closer similarity, and blue means farther similarity.

DEGs with |logFC| > 0.5 were selected for WGCNA, and a scale-free co-expression network was established. The soft-thresholding power β was nine with scale-free *R*^2^ > 0.85 (Supplementary Figure 1). Then DEGs were clustered into ten co-expression modules through the average linkage hierarchical clustering method to ensure that the number of genes in each module is more than thirty. Different modules were represented by different colors, including black, blue, brown, greenyellow, pink, purple, red, tan, yellow, and gray (Figure 1). Genes in the gray module were uncorrelated and excluded from the subsequent analysis. We then calculated the correlation between module memberships and the gene significance for CAD (Supplementary Figure 2). Modules that meet the following two conditions were selected: (1) the correlation coefficient between the module and CAD was >0.5; (2) the correlation coefficient between module memberships and the gene significance for CAD was more than 0.7. According to the above criteria, 193 hub genes in the brown and pink modules were identified for further analysis. These genes were listed in Supplementary Table 1.

**Figure 2:**
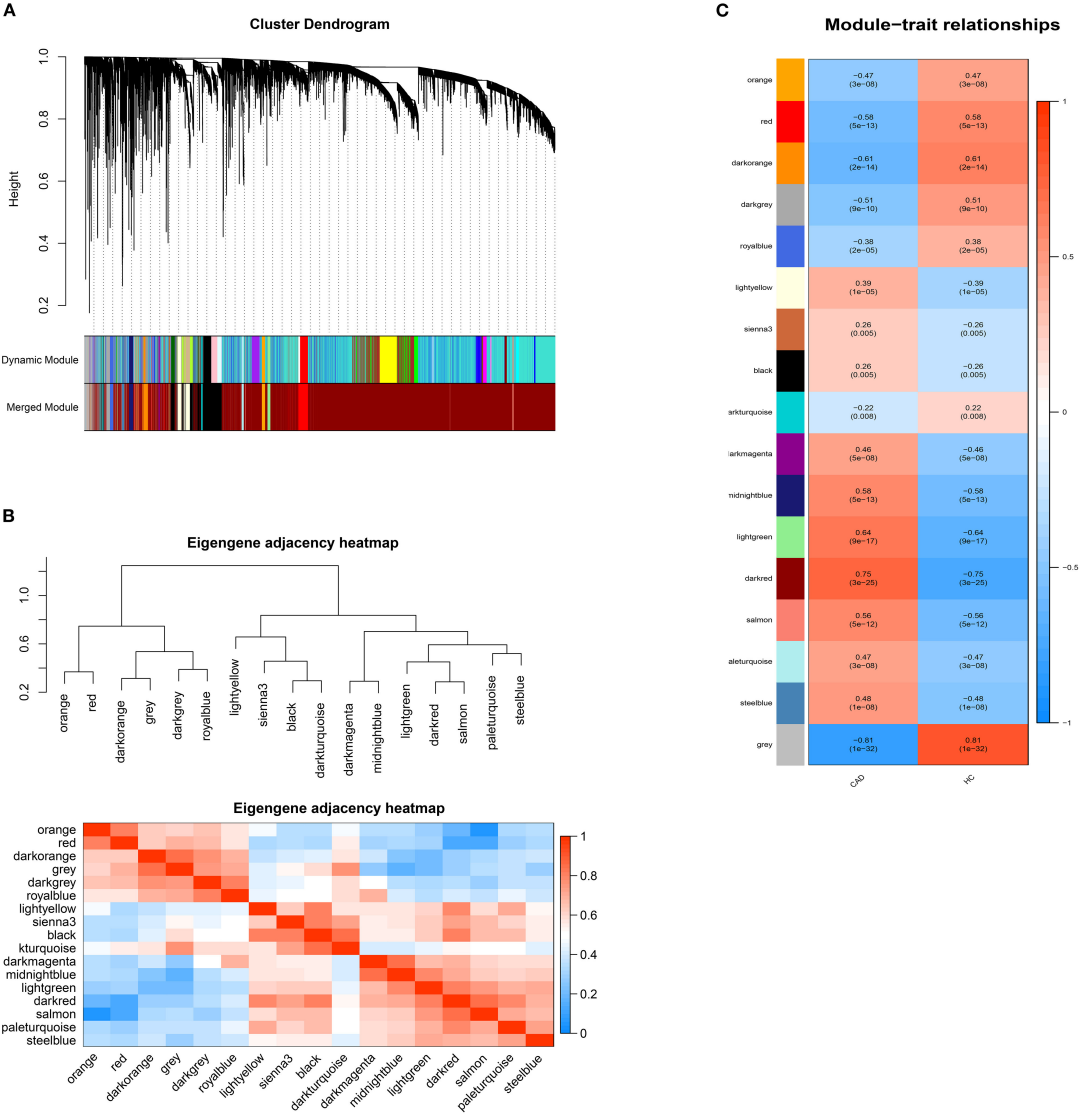
Weighted genes correlation network analysis. **(A)** The dendrogram of differentially expressed lncRNAs. **(B)** The heatmap of module-trait correlations. Blue represents negative correlation, and red represents positive correlation. **(C)** The clustering heatmap between modules. Red means closer similarity, and blue means farther similarity.

DElncRNAs identified by limma package with a *p* < 0.001 were selected for WGCNA. The soft-thresholding power β was ten to ensure scale-free *R*^2^ > 0.85 (Supplementary Figure 3). The DElncRNAs were clustered into sixteen modules with different colors, including black, darkgray, darkmagenta, darkorange, darkred, darkturquoise, lightgreen, lightyellow, midnightblue, orange, paleturquoise, red, royalblue, salmon, sienna3, and steelblue, excluding the gray module (Figure 2). The correlation between module memberships and the gene significance for CAD was also calculated (Supplementary Figure 4). Same as the module selection criteria above, the salmon and lightgreen modules were selected to construct the ceRNA networks.

### Functional Enrichment

**Figure 3:**
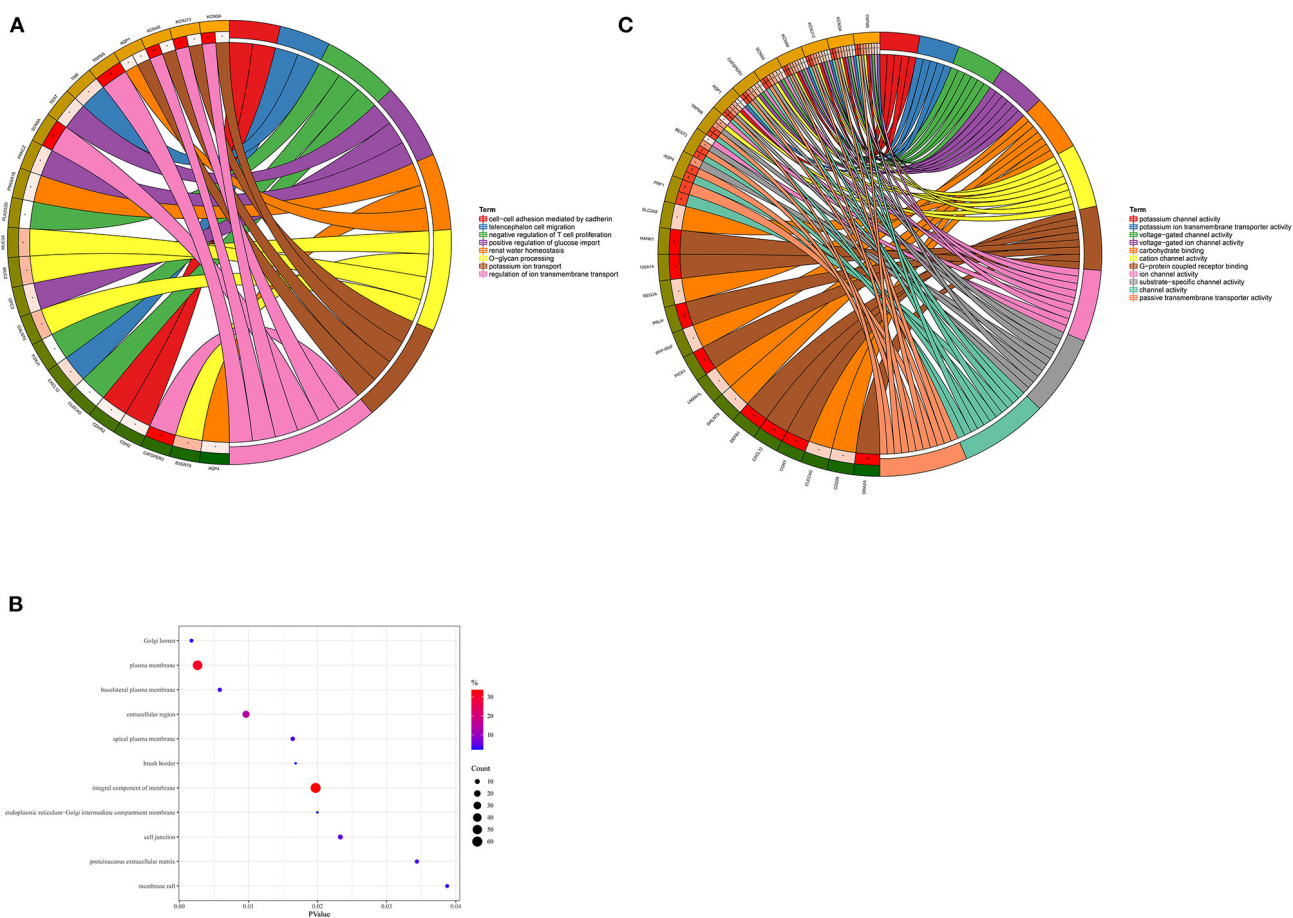
Gene Ontology enrichment analysis. **(A)** Biological process. **(B)** Cellular component. **(C)** Molecular function.

**Figure 4:**
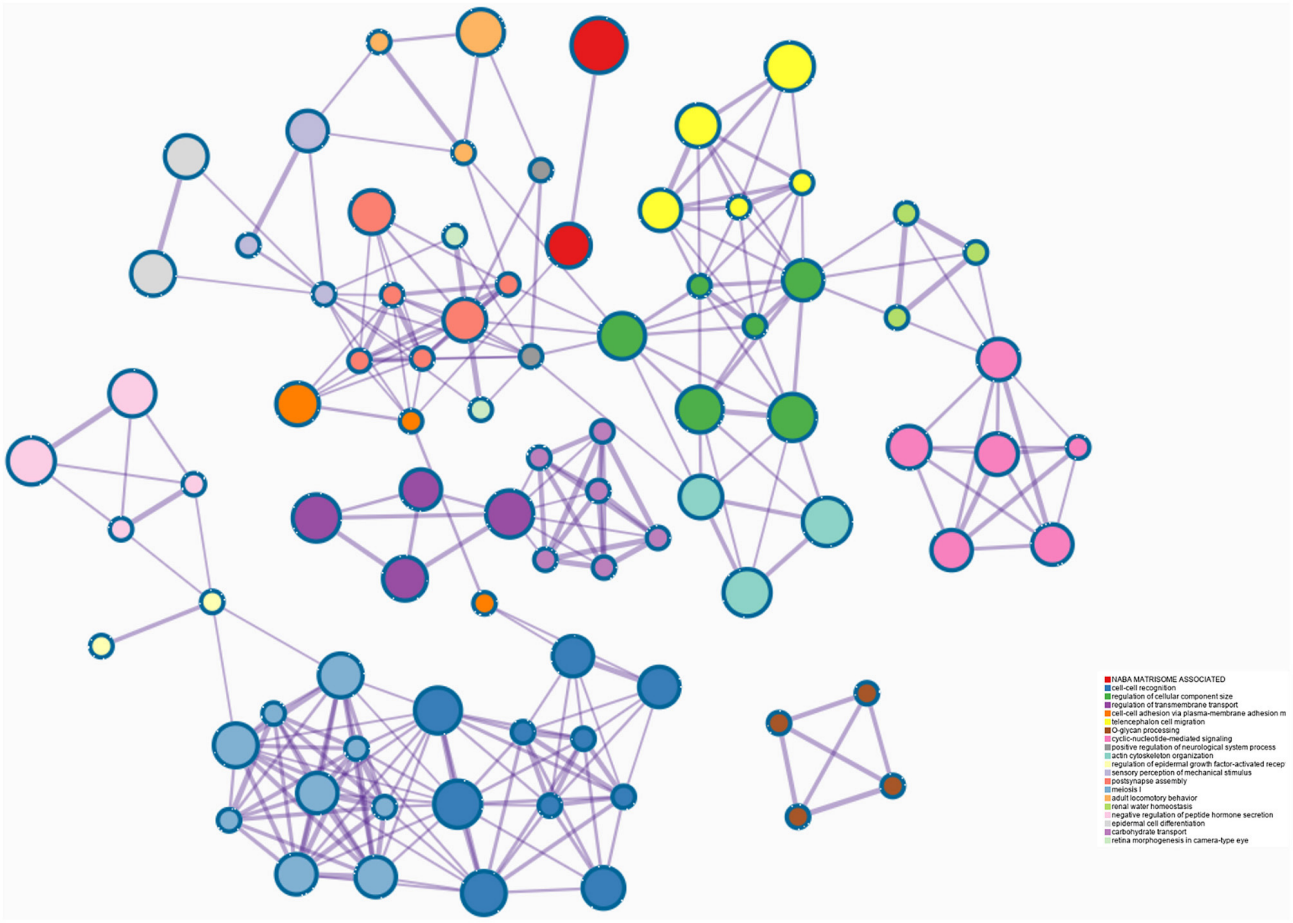
Enrichment analysis using the Meatscape.

193 hub genes in the brown and pink module were subjected to perform functional enrichment analysis utilizing the DAVID and Metascape online tool to investigate the biological effects. The significant enriched GOBPs included regulation of ion transmembrane transport, O-glycan processing, telencephalon cell migration, positive regulation of glucose import, and renal water homeostasis. In addition, golgi lumen, plasma membrane, and extracellular region were significantly enriched in GOCCs. For MF, the most significant entries were G-protein coupled receptor binding, channel activity, and passive transmembrane transporter activity (Figure 3). The results of Metascape demonstrated that hub genes were mainly enriched in the matrisome-associated pathway, cell-cell recognition, regulation of cellular component size, regulation of transmembrane transport, and cell-cell adhesion *via* plasma-membrane adhesion molecules (Figure 4).

### The ceRNA Regulatory Network

**Figure 5:**
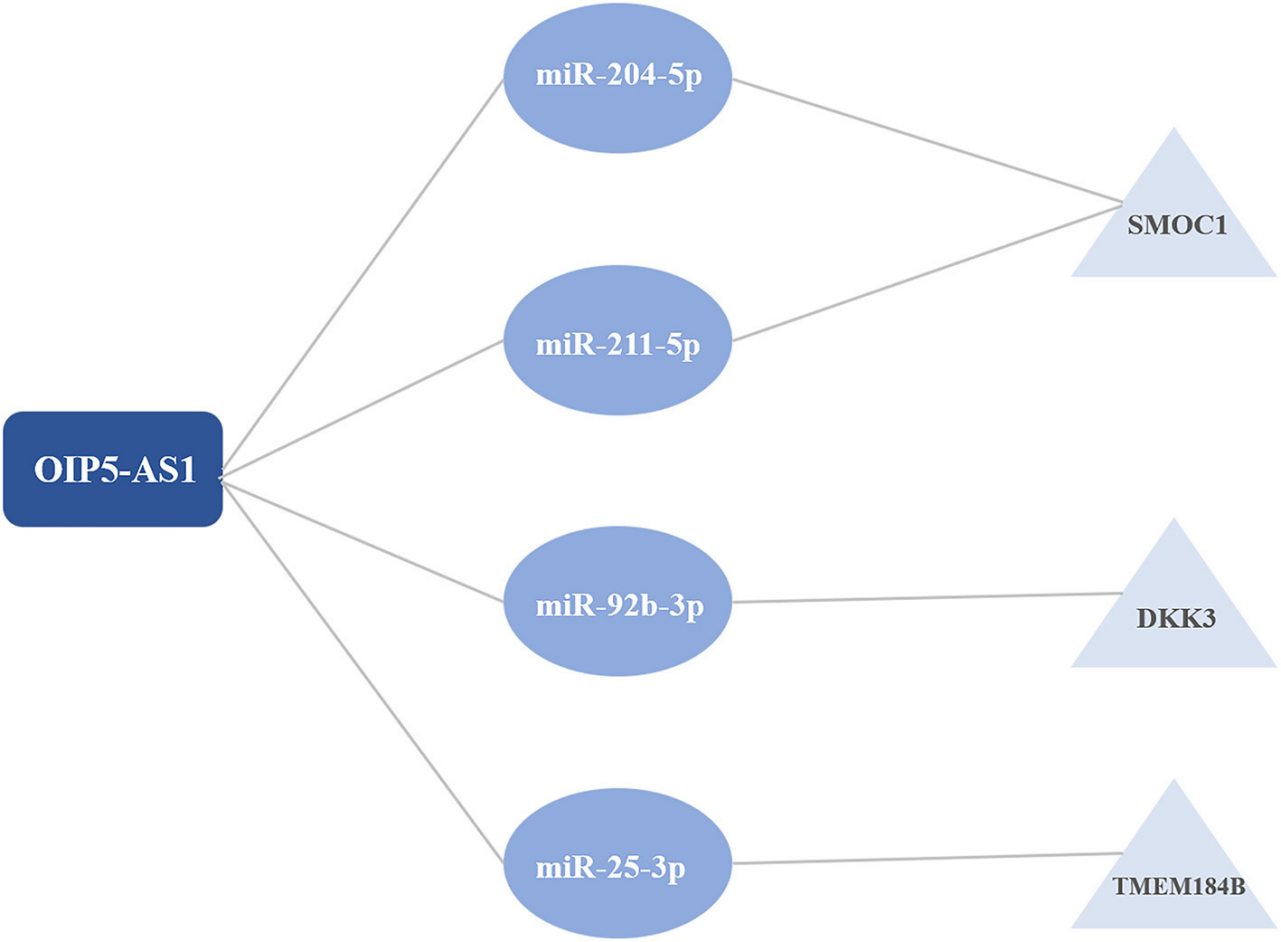
The competitive endogenous RNA (ceRNA) regulatory networks.

The intersection of the online tool miRWalk and three databases, TargetScan, miRDB, and miRTarBase was established to predict the target miRNAs of hub genes in the brown module. A total of seven miRNAs were screened out, including hsa-miR-195-3p, hsa-miR-188-5p, hsa-miR-204-5p, hsa-miR-211-5p, hsa-miR-526b-3p, hsa-miR-92b-3p, and hsa-miR-25-3p. Among them, hsa-miR-195-3p and hsa-miR-188-5p regulate UBE2I; hsa-miR-204-5p, hsa-miR-211-5p, and hsa-miR-526b-3p regulate SMOC1; hsa-miR-92b-3p regulates DKK3 and has-miR-25-3p regulates TMEM184B; has-miR-15b-5p and has-miR-503-5p regulate C1orf21. The starbase database was used to predict interaction relationships between miRNAs and lncRNAs, and the intersection of the predicted lncRNAs and DElncRNAs in modules identified above was established to search for lncRNAs which may play a potential role in the pathophysiological process of CAD. The lncRNA OIP5-AS1 was identified to regulate hsa-miR-204-5p, hsa-miR-211-5p, hsa-miR-92b-3p, and hsa-miR-25-3p. The ceRNA regulatory network was then visualized using the Cytoscape software (v3.8.1) (Table 2; Figure 5).

**Table 2:** The regulatory networks between lncRNAs, miRNAs, and mRNAs.

**lncRNA**	**miRNA**	**mRNA**
OIP5-AS1	miR-204-5p	SMOC1
OIP5-AS1	miR-211-5p	SMOC1
OIP5-AS1	miR-92b-3p	DKK3
OIP5-AS1	miR-25-3p	TMEM184B

### Validation Using qRT-PCR

**Figure 6:**
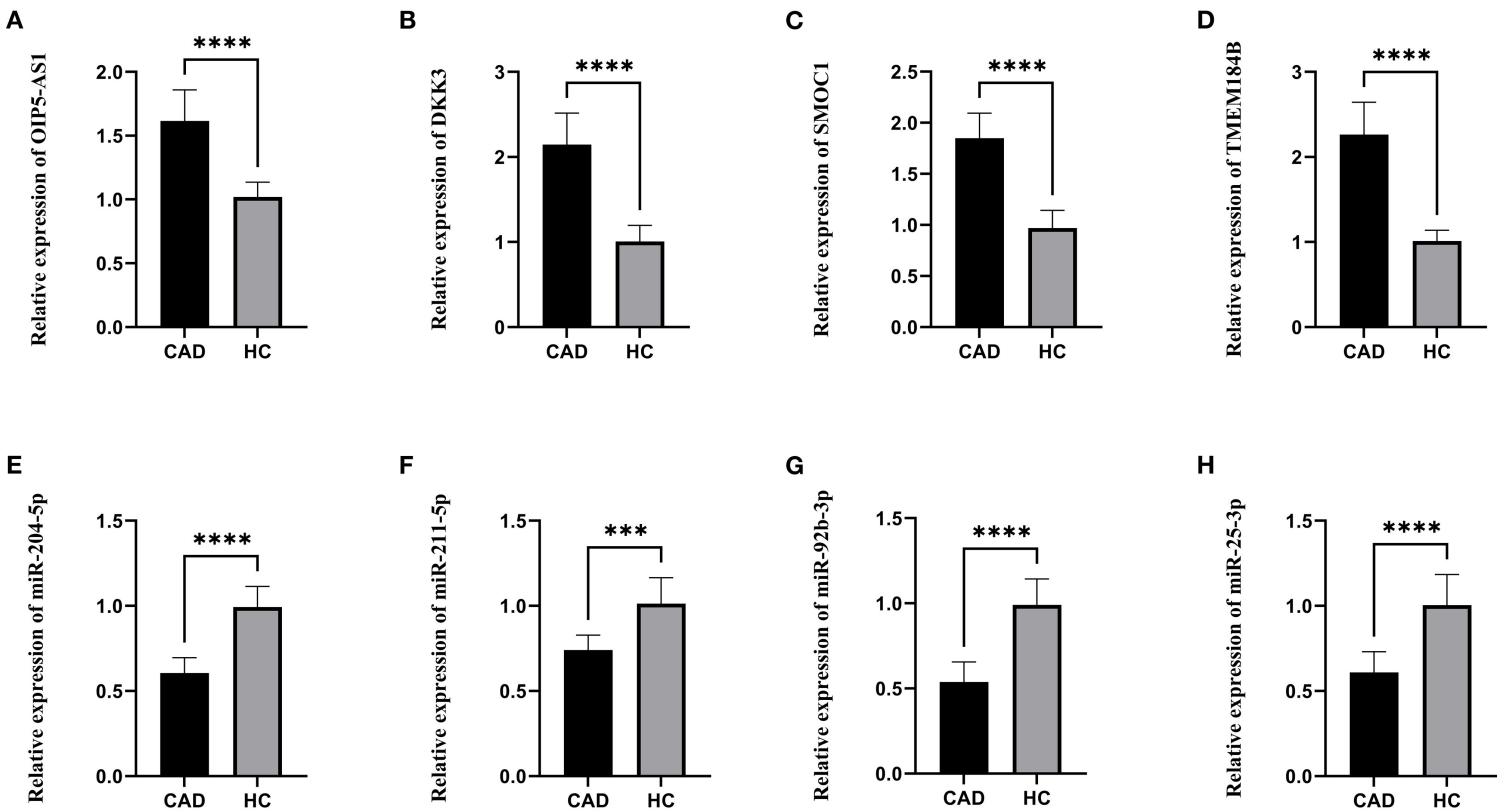
The relative expression levels. **(A)** The relative expression level of OIP5-AS1. **(B)** The relative expression level of DKK3. **(C)** The relative expression level of SMOC1. **(D)** The relative expression level of TMEM184B. **(E)** The relative expression level of miR-204-5p. **(F)** The relative expression level of miR-211-5p. **(G)** The relative expression level of miR-92b-3p. **(H)** The relative expression level of miR-25-3p. CAD, Coronary artery disease; HC, Healthy control. ***p < 0.001, ****p < 0.0001.

The lncRNAs, miRNAs, and mRNAs identified above were verified utilizing qRT-PCR. The results demonstrated that the expression levels of OIP5-AS1, DKK3, SMOC1, and TMEM184B were significantly higher in patients with CAD, while miR-204-5p, miR-211-5p, miR-92b-3p, and miR-25-3p levels were significantly lower, which were consistent with our bioinformatic analysis (Figure 6).

## Discussion

In this study, we downloaded the GSE113079 dataset from the GEO database for bioinformatics analysis. The limma package in the R language was applied to identify the DEGs and DELncRNAs between patients with CAD and healthy controls. Then WGCNA was performed to cluster DEGs and DELncRNAs into different modules and calculate the relationships between modules and CAD. The brown module of DEGs was identified as the key module with the highest correlation coefficient. 188 hub genes in the brown module were selected for functional enrichment analysis. The significant enriched GOBPs included regulation of ion transmembrane transport, O-glycan processing, telencephalon cell migration, positive regulation of glucose import, and renal water homeostasis. The results of Metascape showed that these hub genes were mainly enriched in the matrisome-associated pathway, regulation of cellular component size, and cell-cell recognition. Then three online databases, including TargetScan, miRDB, and miRTarBase, and the online tool miRWalk were used to predict the potential target miRNAs. Nine miRNAs which may regulate hub genes were identified, including hsa-miR-195-3p, hsa-miR-188-5p, hsa-miR-204-5p, hsa-miR-211-5p, hsa-miR-526b-3p, hsa-miR-92b-3p, hsa-miR-25-3p, has-miR-15b-5p and has-miR-503-5p. The Starbase database was used to predict the relationships between seven miRNAs and DElncRNAs in modules selected above to identify potential interactions. The lncRNA OIP5-AS1 was screened out to regulate hsa-miR-204-5p, hsa-miR-211-5p, hsa-miR-92b-3p, and hsa-miR-25-3p. In all, we identified three novel ceRNA networks, including OIP5-AS1 - miR-204-5p/miR-211-5p - SMOC1, OIP5-AS1 - miR-92b-3p – DKK3, and OIP5-AS1 - miR-25-3p - TMEM184B, which have not been studied in CAD before. In the previous study conducted by He et al. (10), they also utilized the GSE113079 dataset to identify the DEGs and DElncRNAs in CAD. However, we applied WGCNA, which can explore the relationships between gene modules and the clinical phenotypes and make results more reliable, to further analyze DEGs and DElncRNAs in the present study and identified the different regulatory nodes. Moreover, He et al. only verified five miRNAs in ceRNA networks, whereas we verified all lncRNAs, miRNAs, and mRNAs in the ceRNA networks using our clinical samples to make our results more credible and worthy of further study in clinical application.

OIP5-AS1 is a long non-coding RNA located on human chromosome 15q15.1, which is involved in regulating cell proliferation (11). Some studies have shown that OIP5-AS1 is related to the pathophysiological process of atherosclerosis. A previous study demonstrated that OIP5-AS1 contributed to the progression of atherosclerosis by targeting miR-26a-5p, and OIP5-AS1 knockdown could promote cell proliferation and reduce apoptosis and inflammatory response (12). A recent integrated analysis identified six key lncRNAs, including OIP5-AS1, whose expression pattern was highly correlated with the disease stage of atherogenesis (13). Another study showed that OIP5-AS1 promoted oxidative low-density lipoprotein induced endothelial cell injury, which may be involved in the pathological process of atherogenesis (14). Moreover, OIP5-AS1 could activate the SIRT1/AMPK/PGC1α pathway by sponging miR-29a to attenuate myocardial ischemia/reperfusion injury (15). OIP5-AS1 is also considered as a carcinoma-related lncRNA in many types of cancer. It was proved to be overexpressed in lung cancer (16, 17), breast cancer (18), osteosarcoma (19, 20), and hepatoblastoma (21), which were related to later cancer stages and metastasis. However, in multiple myeloma and radioresistant colorectal cancer, OIP5-AS1 was downregulated and played an important role in anti-tumor effects (22). In addition, current evidence suggested that OIP5-AS1 was related to osteoarthritis (23), rheumatoid arthritis (24), primary open angle glaucoma (25), and diabetes (26, 27).

The microRNA miR-204-5p and miR-211-5p were predicted to regulate SMOC1. Down-regulation of miR-204-5p was proved to attenuate endothelial cell dysfunction, which was associated with atherogenesis (28, 29). Another study demonstrated that the expression level of miR-204-5p was significantly lower in atherosclerotic plaque tissues and blood samples than in healthy controls. Further studies indicated that miR-204-5p played a crucial role in the growth and migration of human vascular smooth muscle cells by targeting MMP-9 (30). Moreover, mir-204-5p was also involved in the pathophysiological process of aortic valve calcification, which shared many common characteristics of atherogenesis (31, 32). The microRNA miR-211-5p could inhibit cortical neuron differentiation and survival and strengthen oxidative stress in Alzheimer's disease (33, 34). In osteoarthritis, miR-211-5p contributed to chondrocyte differentiation by suppressing Fibulin-4 expression (35, 36). Several previous studies showed that mir-211-5p was also associated with many types of cancer, including renal cancer, hepatocellular carcinoma, breast cancer, and malignant melanoma (37–43). SMOC1 is a protein-coding gene that may play an important role in ocular development (44–47). Recent studies demonstrated that SMOC1 was also associated with Alzheimer's disease (48–50). However, the role of miR-211-5p and SMOC1 in cardiovascular diseases has rarely been studied.

MiR-92b-3p-DKK3 and miR-25-3p-TMEM184B were also identified in our study. Compared with the peripheral venous circulation, the level of expression was lower for miR-92b-3p in the coronary sinus of patients with heart failure (51). The expression level of miR-92b-3p was lower under the hypoxic condition, and it can inhibit proliferation and cell cycle progression in pulmonary arterial hypertension (52). Previous research indicated that miR-92b-3p played a crucial role in vascular smooth muscle cell proliferation by hypoxia (53). Another study showed that it could inhibit cardiomyocyte hypertrophy by targeting HAND2 (54). DKK3 is a member of the Dickkopf family, which is decreased in a variety of cancers serving as a tumor suppressor gene (55). In ApoE-deficient mice, the expression of DKK3 was involved in the pathogenesis of atherosclerosis *via* the Wnt/β-catenin pathway (56). In a prospective population-based study, the expression of plasma DKK3 was inversely related to the 5-year progression of carotid atherosclerosis (57). Serum DKK3 level was also inversely associated with coronary stenosis in a Chinese cohort (58). Another study demonstrated that DKK3 might have a therapeutic effect in reducing intraplaque hemorrhage related to atherosclerotic plaque phenotype (59). These pieces of evidence indicated that DKK3 might play an important role in CAD. The miR-25-3p inhibited coronary vascular endothelial cell inflammation through the NF-kappaB pathway in ApoE^−/−^ mice (60). It was reported that miR-25-3p could promote endothelial cell angiogenesis in aging mice (61). TMEM184B is a protein-coding gene that may activate the MAPK signaling pathway. The role of TMEM184B in cardiovascular diseases has never been reported, which is worth of further research.

Previous WGCNA studies in CAD all focused on the expression of mRNA. To the best of our knowledge, it is the first time that WGCNA was used to analyze the expression of lncRNA and mRNA between patients with CAD and healthy controls and to construct the ceRNA regulatory networks. Nevertheless, there are some limitations to our study. First, the data we acquired was from the public database, lacking clinical trait data. Second, although we identified three novel ceRNA networks in this study, further mechanistic studies should be conducted for a better understanding of the pathological process in CAD.

## Conclusion

In this study, we identified three novel ceRNA regulatory networks, including OIP5-AS1-miR-204-5p/miR-211-5p-SMOC1, OIP5-AS1-miR-92b-3p-DKK3, and OIP5-AS1-miR-25-3p-TMEM184B, using integrated bioinformatics analysis, which were worthy of further study. Our research might provide a novel insight into ceRNA mechanisms in CAD progression.

## Data Availability Statement

The dataset presented in this study can be found at https://www.ncbi.nlm.nih.gov/geo/query/acc.cgi?acc=GSE113079.

## Ethics Statement

The studies involving human participants were reviewed and approved by Medical Ethics Committee of Shanghai Tenth People's Hospital, Tongji University. The patients/participants provided their written informed consent to participate in this study.

## Author Contributions

All authors listed have made a substantial, direct and intellectual contribution to the work, reviewed the final manuscript, and approved it for publication.

## Funding

This study was supported by the General Program of the National Natural Science Foundation of China (No. 81770408).

## Conflict of Interest

The authors declare that the research was conducted in the absence of any commercial or financial relationships that could be construed as a potential conflict of interest. The reviewer JY declared a shared affiliation, with no collaboration, with the authors to the handling editor at the time of review.

## Publisher's Note

All claims expressed in this article are solely those of the authors and do not necessarily represent those of their affiliated organizations, or those of the publisher, the editors and the reviewers. Any product that may be evaluated in this article, or claim that may be made by its manufacturer, is not guaranteed or endorsed by the publisher.
