# Supplementary figures and images for: The Integrative Analysis of Competitive Endogenous RNA Regulatory Networks in Coronary Artery Disease
Source: Front Cardiovasc Med. 2021 Sep 22;8:647953. doi: 10.3389/fcvm.2021.647953 (PMC8492936; doi:10.3389/fcvm.2021.647953)

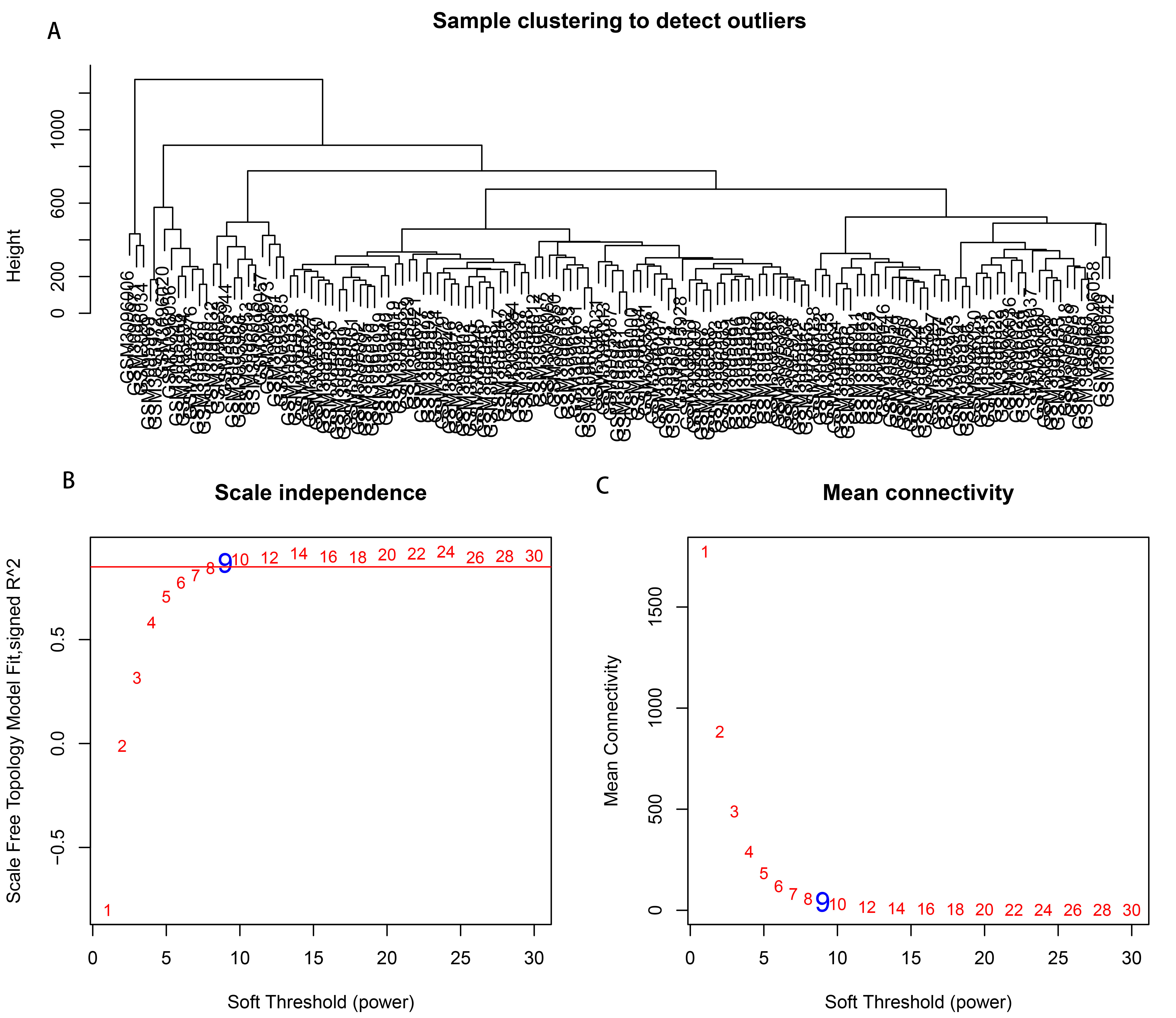

Supplement: Supplementary file 1 [file Image_1.JPEG]

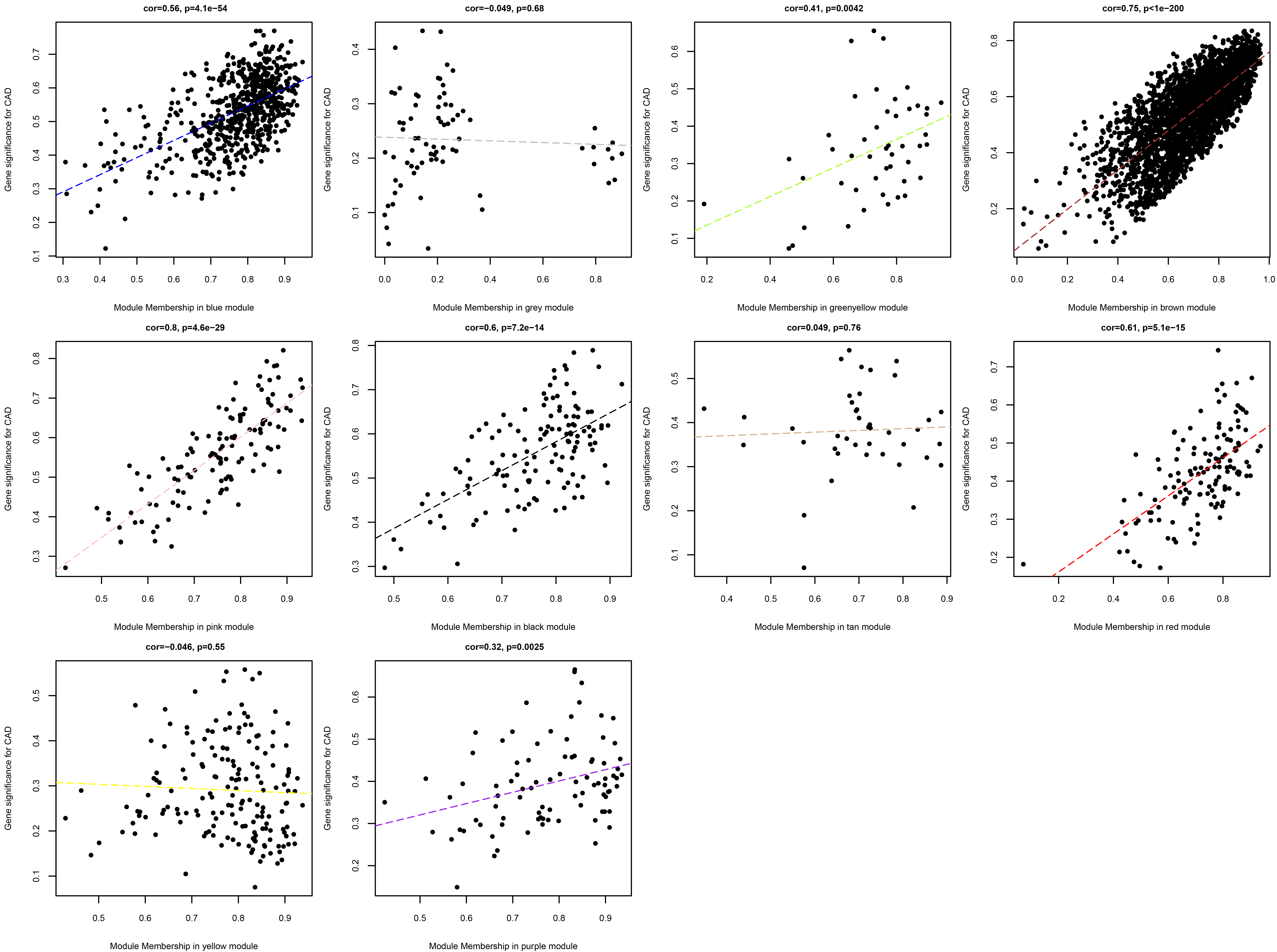

Supplement: Supplementary file 2 [file Image_2.JPEG]

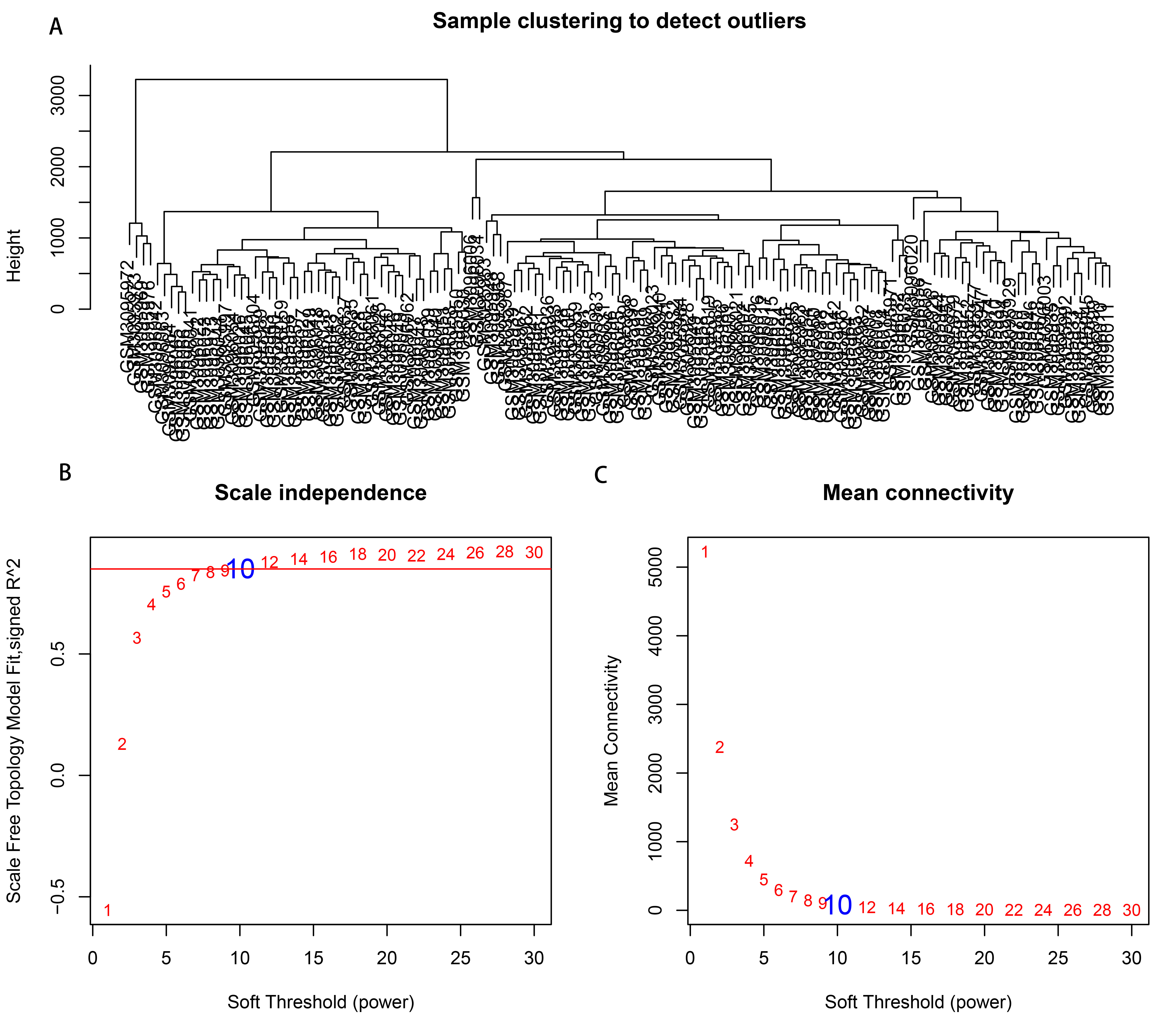

Supplement: Supplementary file 3 [file Image_3.JPEG]
